# Sinoorbital Mucormycosis Associated with Corticosteroid Therapy in COVID-19 Infection

**DOI:** 10.1155/2021/9745701

**Published:** 2021-10-28

**Authors:** Zeinab Mehrabi, Maryam Salimi, Kianoush Niknam, Farzaneh Mohammadi, Hesan Jelodari Mamaghani, Mohammad Reza Sasani, Mohammad Javad Ashraf, Amirhossein Salimi, Mohammad Hassan Zahedroozegar, Zohreh Erfani

**Affiliations:** ^1^Department of Internal Medicine, Namazi Hospital, Shiraz University of Medical Sciences, Shiraz, Iran; ^2^Student Research Committee, Shiraz University of Medical Sciences, Shiraz, Iran; ^3^Department of Surgery, Eastern Health, Victoria, Australia; ^4^Students' Scientific Research Center (SSRC), Tehran University of Medical Sciences, Tehran, Iran; ^5^Department of Radiology, Medical Imaging Research Center, Shiraz University of Medical Sciences, Shiraz, Iran; ^6^Department of Pathology, Shiraz University of Medical Sciences, Shiraz, Iran; ^7^Student Research Committee, Shahid Sadoughi University of Medical Sciences, Yazd, Iran; ^8^Health Policy Research Center, Institute of Health, Shiraz University of Medical Sciences, Shiraz, Iran

## Abstract

**Background:**

Mucormycosis is a rare and invasive fungal infection, affecting almost exclusively immunocompromised individuals. Immunosuppressive effects of corticosteroids which are widely prescribed in COVID-19 patients might be a predisposing factor for opportunistic infections even though the other factors should also be considered. *Case Presentation*. A middle-aged man without any significant past medical history was admitted to the hospital due to a severe COVID-19 infection. He received a high dose of corticosteroids as a part of the treatment. Five days after discharge, he presents with a headache and fever. Eventually, orbital mucormycosis was diagnosed for him and he was treated with antifungal medications.

**Conclusion:**

Opportunistic infections should be considered during the current pandemic of COVID-19, during which corticosteroids are widely prescribed.

## 1. Background

Mucormycosis is a rare, potentially fatal infection caused by fungi from the order Mucorales. [1, 2]. It has various clinical presentations and predominantly affects immunocompromised patients, especially those with diabetes, renal failure, hematological malignancies, and transplant recipients [3, 4]. Because of the high mortality and morbidity rate, prompt diagnosis and intervention are essential [5]. In this case report, we present a male patient with no underlying disease who presented with orbital mucormycosis following hospitalization due to coronavirus disease 2019 (COVID-19) infection. We also aimed to find out the possible association between mucormycosis, COVID-19 infection, and/or medication used to treat them.

## 2. Case Presentation

**Figure 1:**
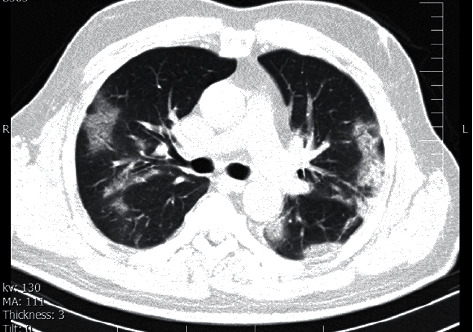
Axial chest CT image without contrast administration showed areas of ground-glass opacities and bilateral consolidations with the predominantly peripheral location.

A 51-year-old man without past medical or social history presented with fever and cough, followed by dyspnea. He was referred to the Khalije Fars Hospital, Busher, and admitted there with the impression of COVID-19 in July 2020. The patient's temperature was 39.4°C, and his pulse was 84/minute. Bilateral fine crackles were auscultated on both lungs. Chest computerized tomography (CT) revealed a patchy ground-glass appearance in both lungs, in favor of COVID-19 (Figure 1). The real-time reverse transcription-polymerase chain reaction (RT-PCR) test was positive for COVID-19.

Laboratory evaluation revealed leukopenia with white blood cell counts (WBC) of 2700 per/*μ*L and lymphocyte count of 864/*μ*L. Additionally, mild thrombocytopenia (platelet: 128000 per/*μ*L) and elevated qualitative C-reactive protein (+3) were detected.

Based on a definite diagnosis of COVID-19, he received dexamethasone 4 mg intravenous (IV) per day along with ceftriaxone 1 gram IV for the prevention of bacterial superinfection. After 5 days of treatment (20 mg dexamethasone totally), the patient (Pt) was discharged with stable vital signs, without leukopenia (WBC: 7200/*μ*L) or any other complications. Prednisolone 25 mg, daily, was prescribed for him.

**Figure 2:**
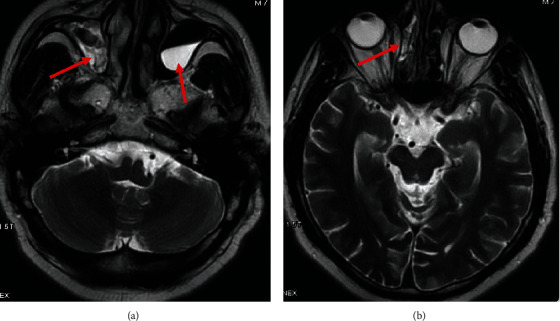
Axial T2-weighted images without contrast administration show (a) mucosal thickening and secretion in the right maxillary sinus and a retention cyst in the left maxillary sinus and (b) mucosal thickening and secretion in the right ethmoid sinus with a lesser degree in the left ethmoid sinus.

Seven days after the hospital discharge (20 mg dexamethasone and 175 mg prednisolone so far), the patient presented with severe headache, but without any significant findings in his physical exam. The brain magnetic resonance imaging (MRI) was in favor of sinusitis (Figure 2). Hence, oral amoxicillin-clavulanate 625 milligram (mg) every 8 hours (Q8h) was prescribed.

**Figure 3:**
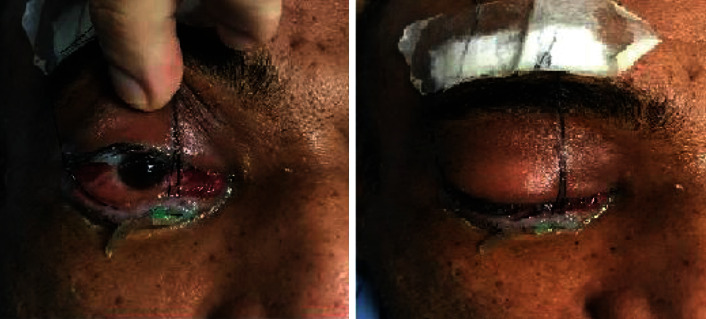
Rt side preorbital edema and redness.

However, his condition did not improve, and he developed right (Rt) side preorbital edema (Figure 3) and redness that eventually spread to the left (Lt) side, so he was referred to an ophthalmologist for better evaluation.

**Figure 4:**
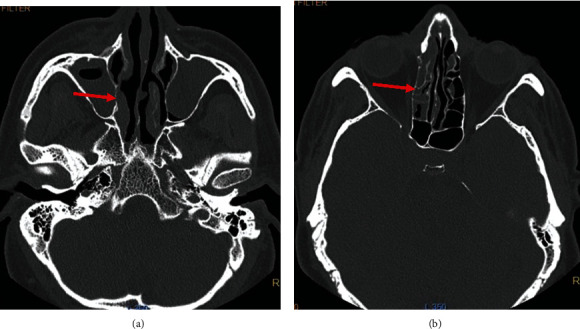
Axial paranasal CT images without contrast administration showing (a) mucosal thickening and secretion in the right maxillary sinus and a retention cyst in the Lt maxillary sinus and (b) mucosal thickening and secretion in the right ethmoid sinus with a lesser degree in the Lt ethmoid sinus.

In his physical examination, tenderness over the sinuses and bilateral preorbital edema were found. The paranasal sinus (PNS) CT scan showed osteomeatal obliteration associated with opacification of bilateral maxillary, ethmoid, frontal, and the Rt sphenoid sinus, suggestive of sinusitis (Figure 4). At this stage, the consumption of oral prednisolone was stopped (in total, 20 mg dexamethasone, and 300 mg prednisolone).

Levofloxacin was prescribed for him with the dosage of 750 mg orally, daily along with artificial tear 1 drop every 4 hours and ciprofloxacin eye drops 1 drop every 8 hours, but his condition worsened; therefore, the patient was referred to an otolaryngologist. The otolaryngologist found a palate lesion in the patient's mouth and took a biopsy from the palate lesion with suspicion of mucormycosis, but no evidence of mucormycosis was found in his pathology. After 5 days, the patient was transferred to Khalili hospital, Shiraz, which is the referral center of otolaryngology and ophthalmology, for anterior orbitotomy due to abscess development. Because of the dangerous condition of the Rt side-eye and the possible disruption of the orbital bones and spread of the infection to the Rt eye, the Lt eye underwent anterior orbitotomy and a cloudy liquid discharge with necrosis and oily component that extended to the orbital wall bone was seen in surgery. The patient was discharged with levofloxacin (750 mg, orally, daily) for sinusitis and prednisolone with a daily dose of 50 mg orally due to suspicion of autoimmune disease.

Three days after his discharge, the patient's condition did not improve, but also, he presented with reduced visual acuity of the Rt eye from 10/10 to 8/10; therefore, the patient has admitted again to Khalili hospital, and in a joint assessment by the ophthalmology and otolaryngology experts, a functional endoscopic sinus surgery (Fess) along with another anterior orbitotomy was performed for the patient. During Fess, necrosis and pussy discharge were found in the paranasal sinuses, and samples from the orbital tissue, nasal cavity, and sinuses were obtained for pathological evaluation and sent to the laboratory of Khalili hospital, affiliated by Shiraz University of Medical Sciences.

**Figure 5:**
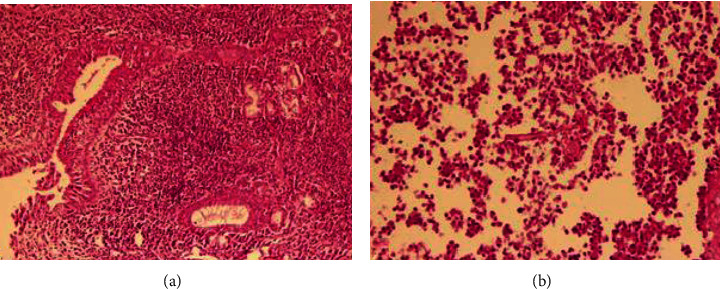
(a) Paranasal sinus mucosa with heavy infiltration of acute and chronic inflammatory cells, H&E stain (×100). (b) Paranasal sinus mucosa with the area of necrosis and hypha which are in favor of the zygomycetes group of fungi, surrounded by polymorphonuclear leukocytes, H&E stain (×200).

Pathology evaluation of the paranasal sinus tissue showed heavy infiltration of acute and chronic inflammatory cells with an area of necrosis and hypha of the mucor fungus surrounded by polymorphonuclear leukocytes, which were in favor of mucormycosis (Figure 5).

The patient was evaluated for human immunodeficiency virus which was negative. Subsequently, antifungal therapy with Amphotericin B lipid complex (200 mg, IV, daily) was started for the patient. The patient was also transferred to Namazi hospital, a referral center affiliated with Shiraz University of Medical Sciences for continuing his antifungal therapy; this hospital has infectious disease specialists.

**Figure 6:**
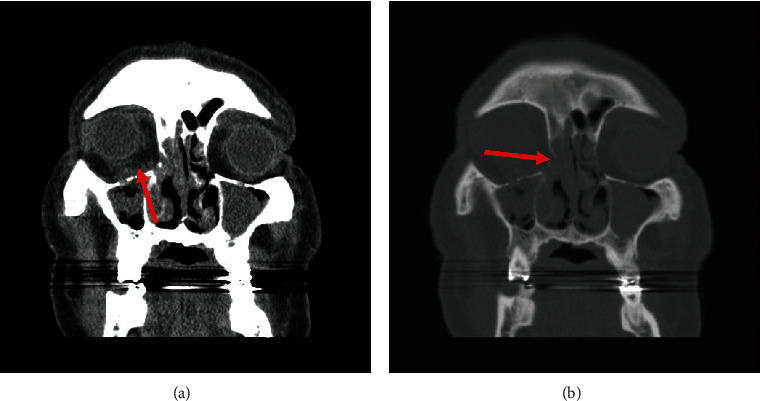
Coronal paranasal CT images without contrast administration. Soft tissue window images: (a) area of increased density in the inferior aspect of right orbit suggestive of orbital inflammation. Mucosal thickening in the right maxillary sinus and a retention cyst in the Lt maxillary sinus was also demonstrated. Bone window image: (b) thinning in the medial aspect of the inferior orbital wall at the right side, suggestive of a small bony erosion (compared to the Lt one).

In Nemazi hospital, visual acuity was reduced to 1/10 and 4/10 in the Rt and Lt eye, respectively. The new PNS CT scan showed progression of the infection (Figure 6).

Therefore, his antifungal treatment regimen was adjusted to Caspofungin (80 mg, IV, daily) and Amphotericin B lipid complex (350 mg, IV, daily) which continued for about one month. Also, for reducing the chance of bacterial superimpose infection, piperacillin-tazobactam (4.5 gr, IV, Q12h) and teicoplanin (400 mg, IV, QD) were administered. During his treatment course, the patient developed hypokalemia, and increased blood urea nitrogen (BUN) and creatinine levels; therefore, Amphotericin dosage was adjusted to 200 mg, IV per day. Also, sixteen days after hospital admission, another Fess with Amphotericin irrigation was done for the patient. He was discharged after 59 days of hospitalization in Nemazi and Khalili hospitals after taking 15700 mg Amphotericin B lipid complex for 56 days, 2400 mg Caspofungin for 30 days, leading to favorable healing. At the time of discharge, the patient had better visual acuity in both eyes (6/10 in the Rt eye and 8/10 in the Lt eye), no orbital or preorbital edema, and tenderness.

## 3. Discussion

COVID-19 was recognized as a pandemic by the World Health Organization (WHO) on March 11, 2020. In the current context, with respect to the increase in the number of infected patients with COIVID-19 as well as the emergence of new species, corticosteroids have been prescribed widely for not only in critically ill patients but also some of the mild to moderate ones regardless of the potential rare adverse complications. In this case report, we described a sinoorbital mucormycosis in an immunocompetent middle age man that was previously treated with high doses of corticosteroid for the prevention of lung fibrosis following COVID-19 infection. In spite of the difficulties of diagnosing the disease, it was successfully managed with medical and surgical interventions.

Corticosteroids have a strong anti-inflammatory effect and are considered to have a role in treating immunologic complications of COVID-19 infection like cytokine storm, lung inflammation, fever, and abnormal laboratory indices of inflammation [5–7]. Observational studies showed that corticosteroid treatment was linked with higher mortality rates and nosocomial infections for influenza and delayed virus clearance for SARS-CoV and MERS-CoV; however, there is limited data regarding SARS-CoV-2 [8]. Also, corticosteroids have been associated with an increased risk of superinfection including bacterial, mycobacterial, and fungal infections [9–12]. Furthermore, several studies have particularly warned about the foregoing adverse effects of corticosteroids in patients with the comorbid disease, but there is little evidence of serious infectious complications in immunocompetent patients without any comorbidities [13–15]. The aforementioned information did not mean to attribute this fungal infection to corticosteroid consumption accurately and purely or did not request corticosteroid elimination. It seeks to warn of the adverse and potentially fatal effects of steroids that may be overlooked because of their many benefits and also to give physicians a comprehensive overview of prescribing the drug.

Mucormycosis is a life-threatening fungal infection that primarily affects patients with immunosuppression [16]. In a study of 101 cases of mucormycosis reported by Lanternier et al., the median number of underlying medical conditions was 2 per patient [17]. Diabetes, hematological malignancies, transplantation, HIV infection, and systemic lupus erythematosus are examples of recognized conditions associated with mucormycosis [16, 18]. The fact that our patient lacked usual comorbidities was associated with a delayed diagnosis of the disease.

As indicated previously, treatment with corticosteroid causes immunosuppression in patients with COVID-19 infection; thus, mucormycosis should be considered as a potential infection affecting them. However, the role of SARS-CoV-2 infection on the cells of the innate immunity (macrophage and neutrophils) should be considered. Our review of the literature indicates that there have been reports of many cases of secondary cases of mucormycosis which is called COVID-19-Associated Mucormycosis (CAM). Those cases have orbital, rhinoorbital, gastrointestinal, and pulmonary involvement [12, 19–22]. In a recent review, a significant percentage of patients with mucormycosis also had a bacterial or fungal coinfection [12]. In a study by Muthu et al., 2,568 cases of CAM are reported which most of them were also affected by diabetes mellitus [23]. Rudramurthy et al. also claimed that uncontrolled diabetes mellitus and inappropriate (high dose or not indicated) corticosteroid use are the major predisposing factors for this surge. Furthermore, a hyperglycaemic state leads to hyperferritinaemia and increased expression of the glucose-regulated protein (GRP-78) in endothelial cells that may help the entry of Mucorales into tissues [24]. The systematic review of literature reveals that there is a significant increase in mucormycosis due to COVID-19, in specific parts of the world, such as India. The reasons for this unprecedented increase remain unknown [25].

The patient was a healthy man that had received high doses of corticosteroids and broad-spectrum antibiotics due to COVID-19. The sign of orbital infection emerged 12 days after the first dose of corticosteroids. Given the lack of any underlying and previous disease or any proven coinfection (e.g., positive urine or blood culture) that usually leads to opportunistic fungal infections such as mucormycotic, we attributed the current infection to the condition called CAM as well as high dose and long duration of corticosteroid administration and its effects on systemic immunosuppression. However, the role of other factors such as iron and zinc metabolism, immunomodulatory effects of COVID-19, endothelial dysfunction which is met in COVID-19 infection, and overexpression of GRP78 should be considered. Our report further highlights the importance of paying attention to the serious complications of COVID-19 disease as well as its treatment of opportunistic infections.

To conclude, physicians should be mindful of the probability of secondary fungal infections in patients with COVID-19 who were managed by corticosteroids even in the absence of any comorbidities. Moreover, the importance of a multidisciplinary approach should be considered.

## Data Availability

All relevant data regarding this case report have been reported in the manuscript. Please contact the corresponding author for any further information.

## Abbreviations

CT: Computerized tomography
RT-PCR: Real-time reverse transcription-polymerase chain reaction
WBC: White blood cell counts
IV: Intravenous
Pt: Patient
MRI: Magnetic resonance imaging
mg: Milligram
BUN: Blood urea nitrogen
Rt: Right
Lt: Left
PNS: Paranasal sinuses
Fess: Functional endoscopic sinus surgery
COVID-19: Coronavirus disease 2019
WHO: World Health Organization.
